# Genomic and Transcriptome Analyses of a Thermophilic Bacterium *Geobacillus stearothermophilus* B5 Isolated from Compost Reveal Its Enzymatic Basis for Lignocellulose Degradation

**DOI:** 10.3390/microorganisms8091357

**Published:** 2020-09-04

**Authors:** Mengmeng Wang, Jiaxi Miao, Xuanqing Wang, Tuo Li, Han Zhu, Dongyang Liu, Qirong Shen

**Affiliations:** Jiangsu Provincial Key Lab of Solid Organic Waste Utilization, Jiangsu Collaborative Innovation Center of Solid Organic Wastes, Educational Ministry Engineering Center of Resource-Saving Fertilizers, Nanjing Agricultural University, Nanjing 210095, China; 2013203040@njau.edu.cn (M.W.); 2017203039@njau.edu.cn (J.M.); 2017203031@njau.edu.cn (X.W.); 2018203039@njau.edu.cn (T.L.); 2018103120@njau.edu.cn (H.Z.); shenqirong@njau.edu.cn (Q.S.)

**Keywords:** thermophilic composting, lignocellulose degradation, comparative genomics, transcriptome

## Abstract

A lignocellulose-degrading strain isolated from thermophilic compost was identified as *Geobacillus stearothermophilus* B5, and found able to secrete considerable amounts of enzymes at optimal temperature (60 °C) and pH (7.5). One circular contig of 3.37 Mbp was assembled from raw data, and 3371 protein-coding genes were predicted. Clusters of orthologous groups (COG) analysis revealed various genes with functions in polymeric substrate degradation, especially for Carbohydrate Active enZymes (CAZymes), such as glycoside hydrolases (GHs) and glycosyl transferases (GTs). Furthermore, the transcriptional responses of B5 at different temperatures—with rice straw provided as the sole carbon source—were analyzed. The results revealed that B5 could resist high temperature by upregulating heat shock proteins (HSPs), enhancing protein synthesis, and decreasing carbon catabolism. Briefly, B5 possesses the ability of lignocellulose degradation, and might be considered a potential inoculant for improving composting efficiency.

## 1. Introduction

Lignocellulosic biomass, which composed of cellulose, hemicellulose, and lignin, are the most widely distributed and renewable organic carbon source on the earth. However, regenerating lignocellulosic feedstock is usually complicated, and the cost is high. Due to these limitations, large amounts of industrial and agricultural lignocellulosic wastes are deposited or burned every year, resulting in an enormous risk of environmental pollution [1]. Thus, technologies to efficiently convert these feedstocks into useful resources are urgently needed. Composting is an economical and socially adopted method, by which organic solid wastes are decomposed and transformed into a stable, humus-like substance. Composting is widely used in urban agriculture, organic farming, landscaping, and other fields. Compost has several benefits when applied to soil, serving as fertilizer or conditioner, and adding vital humus or humic acids. Microorganisms, especially fungi and bacteria, play key roles in the composting process [2]. Although much composting research has focused on fungi, such as *Aspergillus*, *Trichoderma*, and *Penicillium*, bacteria as components of cellulase production strategies has gradually attracted more research attention, due to their resistance to environmental extremes, rapid growth, and expression of multienzyme complexes [3]. These microbes can degrade complex organic substances by secreting high levels of extracellular hydrolytic enzymes into the environment under self-heating, moist, and aerobic environments [4]. Cellulase, xylanase, β-glucosidase, and protease are the key enzymes associated with the composting process, which depolymerize cellulose, hydrolyze xylan, hydrolyze glucosides, and promote N-mineralization, respectively [5]. 

Despite of those advantages, compost may harbor many pathogens, including bacteria, fungi, viruses, and helminths, that can cause infections in humans, other animals and plants. Most obligate and opportunistic pathogens are mesophilic bacteria that can be destroyed during the thermophilic phase, if specific temperature and time criteria are met [6]. According to research by the Environmental Protection Agency (EPA), composting feedstock should be exposed to a temperature over 55 °C for at least three days to ensure the destruction of the pathogens [7]. Therefore, the discovery of thermophilic microorganisms with good potential for composting is attracting increasing interest. In the past few years, much research has aimed at isolating and engineering thermophilic microorganisms for the bioconversion of lignocellulosic substrates. The search for suitable saccharolytic organisms, including ones from the most extreme and remote environments on earth, has led to the discovery of many novel isolates [8]. Among these isolates, those of the genus *Geobacillus*, a versatile, spore-forming, rod-shaped, Gram-positive taxon, can thrive in thermophilic environments. The ability of *Geobacillus* spp. to simultaneously ferment C5 and C6 sugars is very attractive for their application in biomass conversion [9]. Many *Geobacillus* strains are reported to be capable of degrading cellulose or xylan [10], and many industrially important enzymes, including glycoside hydrolases, oxidases, and esterases, originate from *Geobacillus* spp. [11]. 

Whole-genome sequencing affords tremendous insights into the genomic information of enzymes and the secondary metabolites produced by bacteria. Here, we describe a complete genome sequence of *Geobacillus stearothermophilus* B5 and report its transcriptome response at different temperatures by using rice straw as the sole carbon source. The results will convey something meaningful and unique to the readers, and provide insight into the importance and potential of *Geobacillus* spp. in the composting process.

## 2. Materials and Methods 

### 2.1. Screening, Isolation and Identification of the Cellulolytic Strain 

Compost samples were collected from a thermophilic composting heap at a farm in Nanjing, China, which was composed mainly of rice chaff and chicken manure. For the screening of cellulolytic bacteria, ten grams of mixed samples were collected and then vortexed with 90 mL sterile water. The supernatant was serially diluted and then plated on screening medium as described by López-Mondéjar [12] at 60 °C. Cellulose-degrading ability was preliminarily evaluated by qualitative assay following the methods of Teather et al. [13] with cellulose-Congo red agar as the substrate. Morphological, physiological, and biochemical characteristics of the selected strain were analyzed according to the protocols of Bergey’s Manual of Determinative Bacteriology [14]. The morphological features of the cell were observed by using a scanning electron microscope (SEM) after full preparation, as described by Abuga, et al. [15]. The DNA of the selected strain was extracted and amplified by the universal 16S rRNA primers (27F 5′-AGA GTT TGA TCM TGG CTC AG-3′ and 1492R 5′-CGG TTA CCT TGT TAC GAC TT-3′) and *recN* gene primers (forward 5′-CGA TTT GCG GCG ACG ATA-3′ and reverse 5′-TAC ACC ATG CAA AAA CGG TTA C-3′) [16]. The sequences were analyzed for similarities by BLAST against related sequences downloaded from the NCBI database. 

### 2.2. Optimal Culture Conditions and Carbon Source Utilization by Strain B5 

*G. stearothermophilus* B5 was first activated on LB solid plate and then transferred to liquid media with an agitation speed of 170 rpm for 24 h. To determine the optimal culture conditions, growth tests were performed at different temperatures, initial pH values, and NaCl concentrations ranging from 35 to 75 °C, 4.0 to 9.5, and 0% to 4%, respectively. Growth curves were plotted for each of the culture conditions by recording data at one-hour intervals, and the growth rates were determined by evaluating OD600 values. The carbon metabolic characterization of the B5 strain was performed by using the GEN III MicroPlate panel (Biolog, Hayward, CA, USA), which contained 71 carbon sources, along with a positive control and a negative control. Substrate utilization was evaluated colorimetrically concurrent with the spectrophotometric cellular growth measurements. 

### 2.3. Extracellular Protein Extraction and Enzyme Activity Assays

Strain B5 was cultivated in basal salt medium [17] supplemented with 1% (w/v) of one of several carbon sources, including Carboxymethylcellulose sodium (CMC) (Sigma, St. Louis, MO, USA), birchwood xylan (Sigma, USA) and cassava starch (Sigma, USA), to determine enzyme activities. In addition, the B5 strain was cultured in Tryptic Soy Broth medium (Hopebio, Qingdao, China) to detect protease activity. After adding 1 × 10^5^ cfu·mL^−1^ cells to an Erlenmeyer flask, the cells were cultured at 60 °C in an orbital shaker at 170 rpm for 7 days. The bacterial biomass and residual substrates were removed by centrifugation at 12,000 rpm for 10 min, and then filtered through a 0.45 μm membrane, and the supernatant, considered as the crude enzymes, was used in the subsequent experiments. CMCase and xylanase activities were determined by the 3,5-dinitrosalicylic acid (DNS) method according to Liu and Li [18], with CMC and xylan as the substrates, respectively, and α-amylase activity was measured according to Dheeran, Kumar, Jaiswal, Adhikari, and biotechnology [17], with cassava starch as the substrate. One unit of enzyme activity was defined as the amount of enzyme that released 1 µmol of reducing sugars per minute. Protease activity was measured according to Thebti et al. [19], with casein as the substrate. One unit of protease activity was defined as the amount of enzyme that released 1 µmol of tyrosine per minute.

### 2.4. Genome Sequencing, Assembly and Annotation

The genomic DNA was extracted by using the E.Z.N.A.^®^ MicroElute Genomic DNA Kit (Omega Bio-tek, Norcross, GA, USA), according to the manufacturer’s protocol, and quantified by Nanodrop spectrophotometry (Thermo Scientific, Waltham, MA, USA). The purified DNA was sequenced by using PacBio Sequel Single Molecule Real-Time (SMRT) sequencing technology. After genome assembly, the coding sequences (CDSs) were predicted using prodigal software (v2.6.3). Common function annotation was performed by BLAST against the NCBI nonredundant protein (NR), cluster of orthologous groups of proteins (COG), gene ontology (GO), Kyoto Encyclopedia of Genes and Genomes (KEGG), Swiss-Prot, and CAZyme databases. 

### 2.5. RNA Extraction and Transcriptome Sequencing

The strain was first cultured in LB liquid medium at 60 °C and 160 rpm overnight. After removing the supernatant, the cells were washed with sterile distilled water and then transferred to basic medium with 1% rice straw as the carbon source for 10 h. The basic medium contained the following components (in g/L: KH_2_PO_4_, 1.5; Na_2_HPO_4_*·*7H_2_O, 2.5; (NH_4_)_2_SO_4_, 1.5; MgSO_4_*·*7H_2_O, 0.3; CaCl_2_, 0.1; FeSO_4_*·*7H_2_O, 0.005; MnSO_4_, 0.0016; ZnCl_2_, 0.0017; and CoCl_2_, 0.002; pH 7.0). The culture temperature was set at 40 °C (simulating the composting mesophilic phase), 60 °C (simulating thermophilic phase), and 70 °C (simulating the extreme thermophilic phase). Samples collected from all the treatments were immediately frozen in liquid nitrogen for RNA extraction. Total RNA was extracted using the RNA Isolation Kit (Qiagen, Hilden, Germany) and then sequenced for paired-end reads using the Illumina HiSeq 2000 platform (ChosenMed Technology Co., Ltd., Nanjing, China). After quality control, clean reads were retained and mapped to the genome of strain B5 using the TopHat pipeline [20]. The expression of each unigene was estimated by transforming the read density to fragments per kilo base of exon per million mapped reads (FPKM) values. Genes with a threshold of fold change (FC) >2 (or <0.5) and a q-value <0.01 were considered significantly differentially expressed genes (DEGs). 

### 2.6. Statistical Analysis

All biochemical parameters were measured in triplicate and analyzed with the R language (v3.6.1). The relationships between different treatments with respect to the transcriptome data were analyzed using the multidimensional scaling (MDS) method. 

## 3. Results and Discussion

### 3.1. Characteristics of Strain B5

**Figure 1:**
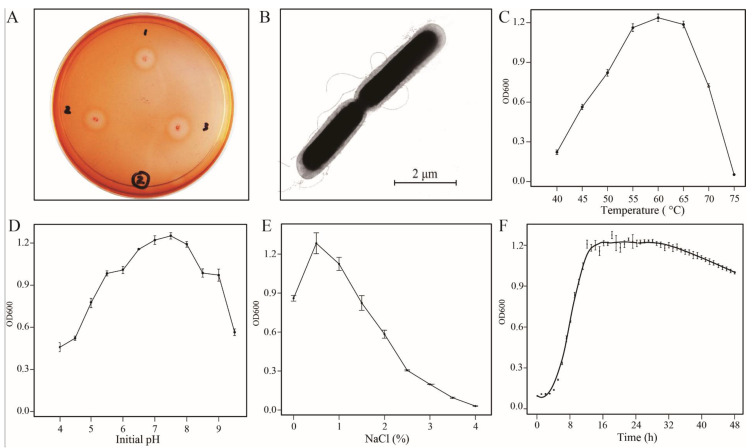
Basic features of *G. stearothermophilus* B5 and the growth curve over 48 h. (**A**) Congo red zone clearing assay. (**B**) Observation of strain B5 under scanning electron microscope. (**C**–**E**) The growth conditions at different temperatures, pH values and salt concentrations for strain B5. (**F**) The growth curve under the optimal culture conditions.

The characteristics of strain B5 were evaluated, and the colony was light yellow, convex, subtransparent, with regular edges, sticky and 1–2 mm in diameter after incubation for 24 h at 60 °C on LB plates (Figure S1). The Congo red assay is a qualitative assay of reducing sugars and commonly used to estimate cellulolytic activity. In the present study, clear zones were observed around the colonies on CMC-minimal media (Figure 1A), indicating pronounced cellulolytic activity. Under the microscope, B5 appeared as motile, Gram-positive, spore-forming, rod-shaped cells, with oval spores located terminally within a swollen sporangium (Figure S1). The size of the cell was 4 to 6 µm in length, 0.5 to 1 µm in diameter, and exhibited peritrichous flagella (Figure 1B). Analysis of the 16S rRNA sequence of this strain indicated that strain B5 is closely related to members of *Geobacillus*. In *Geobacillus*, the *recN* gene has been identified as the most robust marker for assigning new bacterial strains at the species level [21], and homology search revealed that strain B5 is a member of the genus *Geobacillus*, showing the highest similarity (99.71%) to *G. stearothermophilus* DSM 458. Furthermore, phylogenetic analysis indicated that B5 formed a distinct lineage with DSM 458 with 100% bootstrap support (Figure S2). In light of these results and the physiological and biochemical characteristics of this strain (data not shown), B5 was identified as *G. stearothermophilus* B5. The sequence data were submitted to the NCBI SRA database (accession number CP034952). 

The carbon source test results showed that B5 could utilize various carbon sources, including, but not limited to, sugars, amino acids, hexose acids, carboxylic acids, esters, and fatty acids. Positive test results were obtained for D-glucose, sucrose, D-mannose, D-cellobiose, D-mannitol, D-fructose, glycerol, D-turanose, inosine, L-pyroglutamic acid, pectin, glucuronamide, L-malic acid, L-lactic acid, and other compounds (Table S1 and Figure S3). During the composting process, microorganisms are responsible for transforming the organic matter into biomass, CO_2_, heat and humus-like end-products. The broad carbon utilization of B5 revealed that B5 can use a variety of carbon sources during composting. B5 can grow at a broad range of temperatures, from 40 °C to 73 °C, and the optimal temperature for its growth is between 55 °C and 65 °C (Figure 1C). At composting temperatures above 55 °C, many pathogens cannot survive; thus, thermophilic composting is widely adopted for industrial application. Accordingly, the identification of microorganisms with thermoresistance is of much interest. Here, as the B5 strain approached its optimal growth temperature of 55 °C, it was able to efficiently degrade celluloses, hemicelluloses and some other substances. Generally, microbial activity, even for most thermophiles, declines rapidly at temperatures above 63 °C. However, B5 maintained considerable activity at temperatures as high as 65 °C, indicating that B5 might play an important role during thermophilic composting. As one of the critical parameters, the pH values ranging from 5.5 to 9.0 are suitable for most composting microbes, and the optimum pH values are between 4.7 and 8.0 [22]. Interestingly, B5 can survive at pH values ranging from 4.0 to 9.5, and the optimum pH value for growth (Figure 1D) is similar with that for composting efficiency. Furthermore, B5 could survive under NaCl concentrations from 0 to 3.5% (Figure 1E). The growth curve of B5 under the optimal conditions is shown in Figure 1F, and the logarithmic growth period fell within 6 to 12 h. 

### 3.2. Determination of Enzyme Activities 

**Figure 2:**
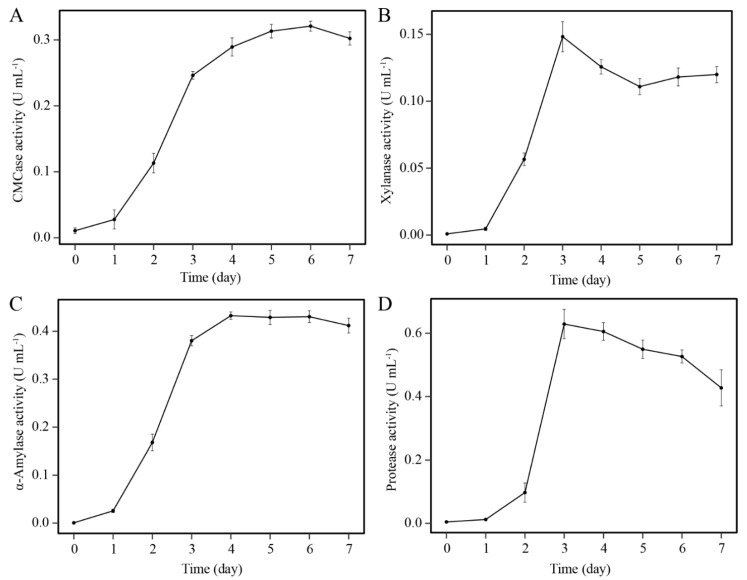
Activities of various enzymes of strain B5 determined by spectrophotometric assays. The time course profile of the activities of CMCase (**A**), xylanase (**B**), α-amylase (**C**) and protease (**D**). The results are replicated three times, and the bar indicates the standard error of the three replicates.

The activities of various enzymes were detected. CMCase increased until peaking (0.32 ± 0.02 U mL^−1^) on the 6th day (Figure 2A) and then decreased until the end of the observation period. Xylanase activity increased sharply, peaking (0.14 ± 0.01 U mL^−1^) on the 3rd day (Figure 2B). The pattern of α-amylase activity was similar to that of CMCase, and its highest value was obtained on the 6th day (0.43 ± 0.02 U mL^−1^) (Figure 2C). Protease activity increased rapidly, similar to xylanase activity, until reaching 0.62 ± 0.03 U mL^−1^ on the 3rd day; it then decreased gradually thereafter (Figure 2D). The biodegradation of lignocellulosic biomass in the composting process requires the synergism of various enzymes including cellulase, hemicellulase, urease, and protease [23]. It is known that filamentous fungi, such as *Aspergillus* spp. and *Trichoderma* spp., can secrete large amounts of extracellular hydrolytic enzymes with high activities; however, the abundances of these mesophilic microbes and their enzyme activities tend to decrease or disappear at the thermophilic phase [3,24]. Interestingly, enzymes produced by thermophilic bacteria are typically more thermostable. Tai et al. [25] found that *Geobacillus* spp. Could secrete CMCase that retained 90% activity after 1 h of incubation at 70 °C. Similarly, in another *Geobacillus* strain, cellulase activity remained 100% stable after 24 h incubation at 60 °C [26]. As a member of *Geobacillus*, B5 could secrete various enzymes with considerable thermostability. Furthermore, a cell-bound effect exists for some enzymes [27], such that the actual efficiency of B5 for degradation of organic might be higher than that measured in cell-free supernatant. According to the previous studies, inoculation with cellulose-degrading microbes can affect the microbial community structure, increase the activities of key enzymes, and accelerate the degradation of cellulose components [28]. Thus, strain B5 might be considered as potential inoculations for lignocellulose composting. A bottleneck for strain B5 is the low yield of thermophilic cells, related to their growth rate; the highest OD600 value of B5 was only approximately 1.2 under the optimal conditions (Figure 1F). In fact, this is also the type bottleneck for most thermophiles. Some measures have been developed to improve cell yield and thus increase enzyme production, such as medium composition, process configuration and the use of specialized equipment [29]. For example, the cellulase production of some *Geobacillus* spp. was increased 2-fold by optimizing the culture conditions with the addition of ammonium sulfate and yeast extract [30]. Thus, more research on B5 is necessary to increase the cell yield and enzyme production. 

### 3.3. Genomic Analysis of G. stearothermophilus B5 and Comparison of COG Categories 

**Figure 3:**
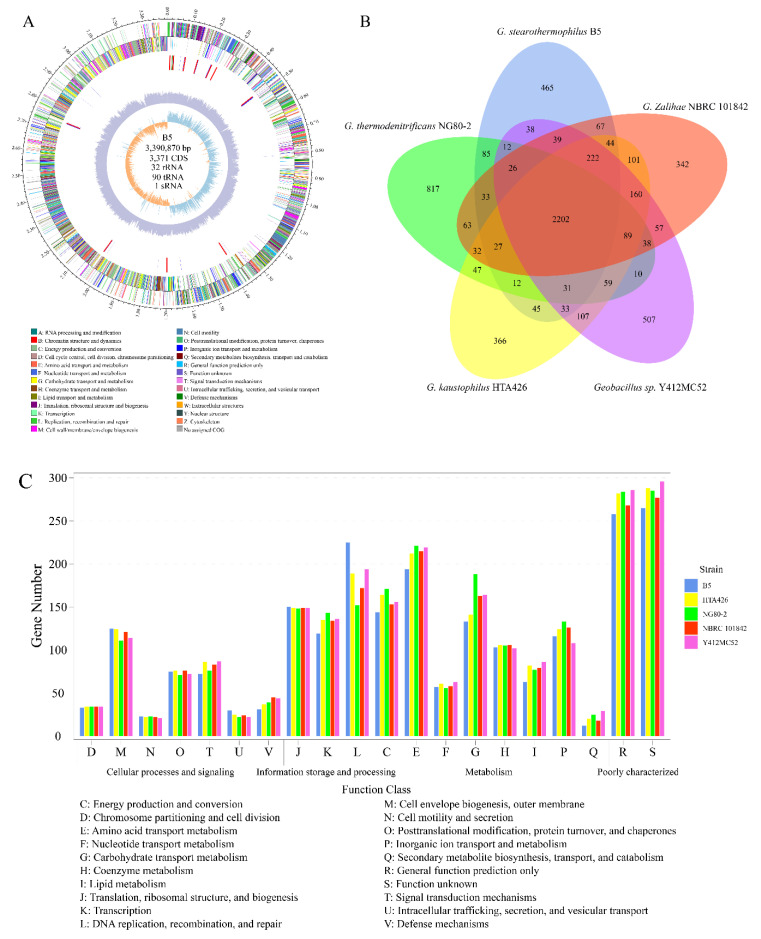
Genomic features of *G. stearothermophilus* B5 and comparisons with other four *Geobacillus* spp. strains. (**A**) Circular map of *G. stearothermophilus* B5 genome features. Circles from the innermost to the outmost represent the following features: G + C skew, G + C content, ncRNA (red represents rRNA, blue represents tRNA and green represents sRNA), clusters of orthologous groups (COG) on backward chains, and COG on forward chains. (**B**) Venn diagram presenting the uniqueness and intersection of encoding genes between *G. stearothermophilus* B5 and four other *Geobacillus* spp. strains. (**C**) Comparison of COG function categories of *G. stearothermophilus* B5 and other *Geobacillus* spp. strains.

The complete genome of *G. stearothermophilus* B5 contains one circular chromosome of 3.39 Mbp with an overall G + C content of 52.46%. No plasmids were detected, and there were 3371 CDSs, 32 rRNAs genes, 90 tRNA genes, and 1 sRNA genes predicted totally (Figure 3A). The gene length to genome ratio was 85%, and the intergenic-region length to genome ratio was 15%. Genes were then annotated with different databases as follows: COG (2472), GO (2363), NR (3342), Swiss-Prot (2617), KEGG (1797), and CAZyme (100). Strain B5 was compared to the other four *Geobacillus* strains that were previously reported to exhibit considerable cellulase or hemicellulase production ability. The genome features of the five strains are presented in Table 1. The numbers of orthologous genes between strain B5 and the other four *Geobacillus* strains were 2615 (HTA426), 2414 (NG80-2), 2417 (NBRC 101842), and 2602 (Y412MC52). The core genome of the five strains consisted of 2202 orthologous genes, and the pangenome consisted of 6175 genes, among which 465 genes were unique to strain B5 (Figure 3B). Conserved genes and gene pools are often used to evaluate the variation among genera. Zhang et al. [31] compared the genomes of five *Bacillus* strains (four *Bacillus amyloliquefaciens* strains and one *Bacillus subtilis* strain). The genes pools consisted of 5643 genes, and 73.9% of them were conserved among the five *Bacillus* strains, which indicated low variation in *Bacillus amyloliquefaciens*. In this study, comparison of the five *Geobacillus* strains revealed that 65.3% of the genes were conserved, and 6175 gene pools were observed, indicating high variation among the *Geobacillus* strains. These variations might be due to the strains’ thermophilic environments.

In strain B5, 2472 genes were annotated to 1581 COGs, and all available CDSs from the five *Geobacillus* strains were assigned to 20 COG functional categories (Figure 3C). There were few differences in most of the cellular processes and signaling categories (D to V). This finding might be because the five strains belong to the same genus, and the major functional models are conserved. The major differences among these five strains were observed in metabolism categories (C to Q), especially carbohydrate transport and metabolism (G). To further investigate the potential roles of strain B5 during composting, specific COGs involved in carbon catabolic functions were analyzed. Amino acid transport and metabolism function and carbohydrate transport and metabolism function represented 10.76% and 7.24% of the COG categories, respectively (Table S2). With respect to amino acid transport and metabolism function, the top five abundant COGs were permeases of the major facilitator superfamily (COG0477), permeases of the drug/metabolite transporter superfamily (COG0697), aminotransferase (COG0436), deacetylase (COG0624), and lyase (COG0346); whereas the top five abundant COGs for carbohydrate transport and metabolism were permeases of the major facilitator superfamily (COG0477), permeases of the drug/metabolite transporter superfamily (COG0697), deacetylase (COG0726), glycosidase (COG0366), and phosphotransferase system IIC component (COG1263). COG0477 participates in encoding permeases of the major facilitator superfamily, which could catalyze the transport of several types of substrates, including carbohydrates, lipids, peptides, nucleotides, and some other molecules, under thermophilic conditions [32]. COG0366 is versatile; it could not only encode glycosidases responsible for the release of aromatic compounds [33], but also α-amylase, which could destroy the alpha bonds between long-chain polysaccharides, such as glycogen and starch [34]. The COG analysis indicated that strain B5 has strong potential for degrading proteins and carbohydrates during composting. The extensive diversity of gene functions revealed considerable potential of *G. stearothermophilus* B5 for organic substance decomposition in composting systems.

### 3.4. CAZyme Family Analysis of the Genome of Strain B5 

CAZymes can break down, create and rearrange oligo- and polysaccharides and play an important role in bacteria and are vital for optimizing biomass degradation [35]. The degradation capacity of strain B5 during the composting process was revealed through the gene annotation against the CAZyme database. Strain B5 encoded 100 CAZymes that were unevenly distributed among glycoside hydrolases (GHs, 29.0%), glycosyl transferases (GTs, 36.0%), carbohydrate esterases (CEs, 20.0%), auxiliary activities (AAs, 4.0%), and carbohydrate-binding modules (CBMs, 11.0%) (Figure S4). The GH and GT family members comprised the largest proportion and fulfill vital functions in the cleavage of polymeric substrates [36].

The GH family enzymes could hydrolyze the glycosidic bond between two carbohydrates or a carbohydrate and a noncarbohydrate moiety. Under thermophilic conditions, GH members of strain B5, including cellulase (GH1, GH3, GH31), amylase (GH13), and chitinase (GH18), together with some kinds of peptidoglycan hydrolase and oligosaccharide-degrading enzymes, were significantly upregulated (Table S3). Genes encoding α-amylase (EC 3.2.1.1), which is considered a crucial amylase, were widely detected in strain B5. The biomass degradation pathways that GH families in strain B5 participate in include glycolysis (ko00010) and starch and sucrose metabolism (ko00500). The GH1 members encoded by strain B5 are 6-phospho-β-glycosidases; these have been found to be extremely thermostable, losing almost no activity after incubation at 60 °C for 7 days, and they could utilize cellobiose [37]. One GH4 member encoding 6-phospho-α-glucosidase and one GH5 member encoding endo-1,4-β-glucanase, also have obvious effects in cellulose degradation. Furthermore, strain B5 contained a higher number of ten GH13 family genes, which participate mainly in starch hydrolyzation [38]. Some CEs were detected in strain B5, which exhibit potential in deacetylating xylan and xylooligosaccharide. A CE3 previously discovered in *T. reesei* could encode acetyl xylan esterase, thus enhancing the solubilization of xylans [39]. Moreover, a CE7 from *Thermoanaerobacterium* spp. was confirmed to degrade xylan [40]. These genes in strain B5 may be important for the biodegradation of cellulose and hemicellulose. Six CE4 members associated with the destruction of plant polysaccharides were also detected in strain B5. These CE4 members were all acetyl xylan esterases, which could catalyze the deacylation of galactoglucomannan and acetylated manno-compounds. The CE4 family also possesses peptidoglycan N-deacetylates that have the ability to degrade chitin [41]. Three AA4 family members, including vanilly-alcohol oxidases (VAO), were detected, the function of which is to catalyze the conversion of multiple phenolic compounds bearing side chains at the para position of aromatic rings [42]. As the vital components of CAZymes, glycosyl transferases could catalyze the transfer of sugar moieties from activated donor molecules to specific acceptor molecules. Thirty-one GTs were detected in B5, predominantly GT4 and GT2. GT4 and GT2 family members represent approximately 50% of all glycosyl transferases, and might be the original members from which other GT families evolved [43,44]. The GT4 and GT2 families could catalyze various reactions, including some key steps in N-glycosylation pathways. GT35 family genes were also detected in strain B5. These genes could catalyze the phosphorolysis of specific glycosidic bonds within maltodextrins by removing the nonreducing glucosyl residues of linear oligosaccharides [45]. The CAZymes identified in this study indicated that strain B5 possesses considerable potential for metabolizing some recalcitrant and readily degradable biomass. Our results also provide genetic evidence of both strong hydrolytic and transglycosylytic capabilities of strain B5, which harbors various kinds of CAZyme genes.

### 3.5. Global Analysis of Transcriptome and DEGs

**Figure 4:**
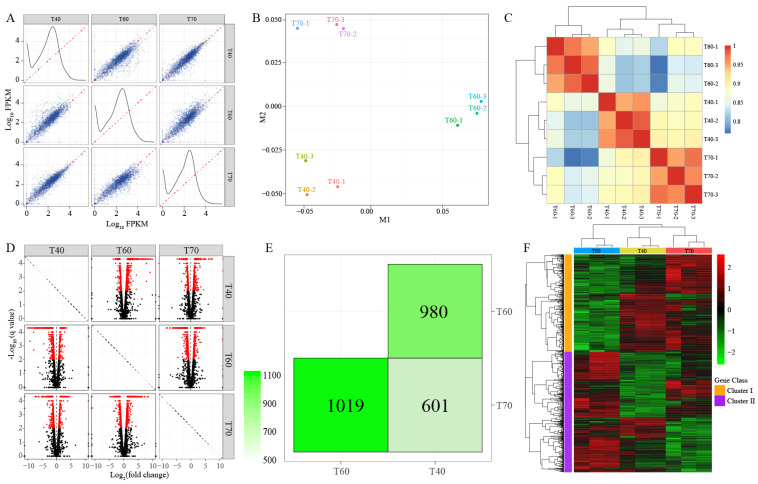
Global analysis of the transcriptome data from different treatments. (**A**–**C**) The distribution and correlations of the fragments per kilo base of exon per million mapped reads (FPKM) values of different treatments shown by scatterplots, MDS and heatmap. (**D**,**E**) Differentially expressed genes (DEGs) among treatments. (**F**) Hierarchical clustering analysis of gene expression profiles with all the DEGs.

The raw data were processed according to the TopHat2-Cufflinks workflow [46], and the expression of all unigenes was obtained, expressed as FPKM values. The FPKM distribution and relationships among different treatments are shown in Figure 4. MDS method was applied to identify the sources of variability within the data. An obvious divergence was observed among different treatments, and the repeatability of the three biological replicates was high (Figure 4B). The results were verified by the Spearman coefficient of correlation among the nine data sets, which shared a value of greater than 0.93 within the same treatment (Figure 4C).

DEGs were detected to identify the temperature-responsive genes between treatments, and a diagram was constructed to visualize the effects of treatment on DEGs (Figure 4D,E). There were 980, 1019 and 601 DEGs between the pairs of treatments. Interestingly, 171 DEGs were shared among all treatments; for most of these, their expression level decreased as temperature decreased or increased to the limits (Table S4). Figure 4F showed the expression differences and their clustering of all DEGs. The DEGs were divided into two groups: in group I, most genes were expressed at a very low levels at T60, and there were many differences between T40 and T70; in group II, almost all the genes were highly expressed in T60, and the DEGs in T40 and T70 showed small differences. 

### 3.6. Metabolism Characteristics of Strain B5 under Mesophilic Conditions

The metabolic systems of microbes are appropriately regulated, especially in nonoptimal environments. The translation and synthesis of proteins are energy-consuming processes and can be expected to be limited to some extent under extreme conditions. The isopropylmalate synthase (gene2488), isopropylmalate dehydrogenase (gene2487), and aminotransferase (gene2492) genes were significantly upregulated in the T40 treatment, compared with the T60 treatment (Table S5). These are key enzymes associated with leucine biosynthesis and identified as playing critical roles in the “valine, leucine and isoleucine biosynthesis” and “valine, leucine and isoleucine degradation” pathways. The expression of branched-chain amino acid transporters (gene3091) belonging to the ABC transport system was also upregulated in T40. These transporters participate mainly in the transportation of branched-chain amino acids (such as leucine) into the cell or their assimilation in the cell. The genes involved in the degradation of branched-chain amino acids, such as branched-chain alpha-keto acid dehydrogenase (gene0976, 2204), were downregulated in the T40 treatment, which illustrated that strain B5 might enhance the biosynthesis of leucine and inhibit its degradation at the mesophilic phase of composting. It has been reported that the D-amino acids (D-Leu, D-Met, and D-Phe) could regulate the synthesis of peptidoglycan [47]. Many bacterial cell walls contain large amounts of peptidoglycan, especially those of Gram-positive bacteria. Thus, the downregulation of UDP-N-acetylmuramoyl-L-alanyl-D-glutamate-2,6-diaminopimelate ligase (gene1033), which is involved in cell peptide synthesis, might result in the inhibition of cell wall synthesis in strain B5. The synthesis of D-amino acids might be a common strategy of bacteria for adapting to nonoptimal environments. In strain B5, the uptake and synthesis of leucine might help compensate for the downregulation of genes related to peptidoglycan synthesis. 

Aromatic amino acids, including tryptophan, phenylalanine and tyrosine, are necessary in all microorganisms for primary metabolism and are produced mainly via the shikimate pathway [48]. In the T40 treatment, the expression of chorismate mutase genes (gene2048, 2622) did not differ from that in T60, whereas anthranilate phosphoribosyl transferase (gene2045) and a pyridoxal phosphate dependent enzyme (gene1050) were upregulated. These findings indicated that the synthesis of phenylalanine and tyrosine might not be affected at the composting mesophilic phase, while the synthesis of tryptophan is strengthened. The above results are supported by the upregulated expression of tryptophan-tRNA ligase (gene0160), which might be a signal of increasing demand for tryptophan. Furthermore, the expression of phosphoglycerate dehydrogenase (gene2090) and phosphoserine aminotransferase (gene0536) was upregulated in T40. These two enzymes are key enzymes during serine biosynthesis, and serine can serve as a precursor for tryptophan synthesis [49]. In addition, the upregulated pyridoxal phosphate enzyme (gene1050) might aid the conversion of serine to tryptophan. The synthesis of tryptophan of strain B5 was upregulated to allow adaption to the low temperature and survival in the composting mesophilic phase. Similarly, the synthesis and uptake of tryptophan was found to increase in *Saccharomyces cerevisiae* under cold stress, which enhanced its tolerance to low temperature [50].

### 3.7. Heat Shock Proteins (HSPs) and Enrichment Analyses of Strain B5 under Extreme Thermophilic Conditions

Strain B5 was isolated from thermophilic composting conditions and able to survive at 70 °C or higher temperatures. Thus, it is certainly worth understanding its internal heat shock mechanism, which might be the prerequisite of B5 strain to secrete extracellular enzymes. Heat shock proteins perform chaperone functions by stabilizing proteins to ensure correct folding or help refold denatured proteins to protect the cell from heat stress [51]. The expression changes of heat shock proteins in the T70 treatment relative to the T60 treatment are presented in Table 2, all of which were at least 2-fold increases. Some HSPs are responsible for unfolding insoluble protein aggregates or serving as cofactors of Hsp70, such as *clpB* (gene0701), *clpX* (gene2482), *clpP* (gene1200), and *dnaJ* (gene2328). Hsp33 (gene0064) and *dnaK* (gene2329) are class I heat shock proteins (chaperonin) and function in protein folding and unfolding; they, thus, confer thermotolerance to cells exposed to extreme stress. *grpE* (gene2330) was discovered in strain B5, which serves mainly as a cofactor of *dnaK*. It appears that these genes play pivotal roles in allowing strain B5 to survive in the thermal environment. 

**Figure 5:**
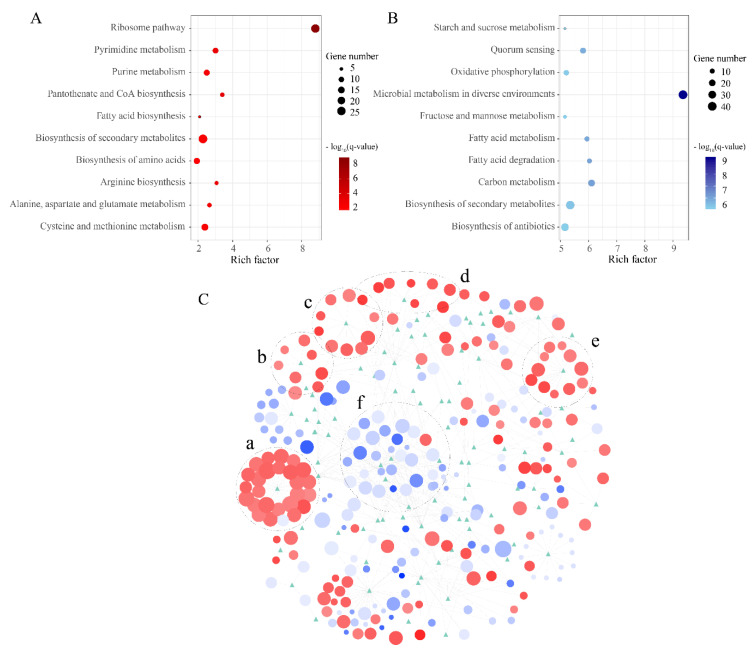
Kyoto Encyclopedia of Genes and Genomes (KEGG) pathway analysis of DEGs between the T70 and T60 treatments. (**A**) Enrichment analysis of upregulated genes (T70 vs T60). (**B**) Enrichment analysis of downregulated genes (T70 vs T60). The x-axis presents the enrichment factor, and the y-axis shows the name of the KEGG pathway; dot size represents the number of associated genes, and color indicates the –log_10_(q-value). The top 10 enriched GO terms are shown in the figure. (**C**) Protein-protein interaction based on the KEGG pathway analysis of differentially expressed genes between the T70 and T60 treatments. The nodes show proteins are marked as circles and KEGG categories are marked as green triangles; edges are protein interactions defined by the KEGG database. Circle size indicates the expression level in the T70 treatment, and color indicates the fold change (red denotes upregulated, blue denotes downregulated) of expression in the T70 treatment relative to the T60 treatment. The black dotted circles (a) is the ribosome pathway, (b) is pantothenate and CoA biosynthesis, (c) is cysteine and methionine metabolism, (d) is arginine biosynthesis, (e) is pyrimidine metabolism, and (f) is some metabolisms related to carbon source, including starch and sucrose metabolism, fructose and mannose metabolism, and fatty acid metabolism.

Other metabolic changes in strain B5 when confronted with high temperature were also investigated. To investigate how this strain responds to heat stress, the DEGs between the T70 and T60 treatments were subjected to KEGG enrichment and protein-protein interaction analyses (Figure 5). The most significant upregulated pathway was ribosome (eco03010, q-value = 1.63 × 10^−9^), covering 33 genes. The participating genes encode various ribosomal proteins (Table S6), including 30S ribosomal protein S3 (gene0114) and 50S ribosomal protein L13 (gene0141). Protein S3 and protein S4 (gene2613) could encircle mRNA when entering the ribosome, and play an important role in mRNA helicase processivity [52]. Protein L13 is very important during the early stage of 50S assembly [53]. The above results were supported by the GO enrichment analysis, in which the top three upregulated enrichment terms were all related to ribosome synthesis (GO:1990904, 0005840, 0003735). All of the results suggested that protein synthesis strongly increased under high temperature to increase heat-resistance capacity. These findings are in contrast to the performance of the thermolabile strain *S. cerevisiae*. In *S. cerevisiae*, the expression level of ribosomal proteins was significantly downregulated when facing heat stress [54]. Different microorganisms showed different strategies under high temperature stress. This difference might reflect that in strain B5, many of the upregulated proteins, especially some key enzymes, are related to cell survival under heat stress. Furthermore, the pantothenate and CoA biosynthesis (eco00770), arginine biosynthesis (eco00220), and pyrimidine metabolism (eco00240) pathways were significantly upregulated under high temperature. Unlike the genes in the ribosome pathway, these genes participate in carbon metabolism (eco01200), fatty acid degradation (eco00071), fructose and mannose metabolism (eco00051), starch and sucrose metabolism (eco00500), biosynthesis of secondary metabolites (eco01110), and some biological process- and cellular metabolic process-related pathways, and were all downregulated to various degrees. These results suggested that the decreased catabolism of carbon could save energy, thus, enhanced the resistance to high temperature.

## 4. Conclusions

An efficient lignocellulose-degrading strain was isolated from thermophilic compost and identified as *G. stearothermophilus* B5. Whole-genome analysis of B5 and comparative analysis provided genomic information and revealed the considerable ability of this strain to degrade lignocelluloses, thus indicating its potential application in agricultural waste management and related fields. In addition, this study explored the responses of B5 at different composting phases via transcriptome analysis. The present work will strengthen the genomic aspect of exploiting bacteria for efficient thermophilic composting.

## Figures and Tables

**Table 1:** Genomic features of *G. stearothermophilus* B5 and comparison with four other reported cellulolytic *Geobacillus* spp. strains.

Feature	B5	HTA426	NG80–2	Y412MC52	NBRC 101842
Size (bp)	3,390,870	3,592,666	3,608,012	3,673,940	3,539,687
GC content (%)	52.46	51.99	48.85	52.31	51.9
Number of contigs	1	2	2	2	164
Protein-coding genes	3371	3546	3554	3596	3515
Mean gene length (bp)	851	861	853	869	849
Percent of coding region (%)	84.60	84.98	84.02	85.06	84.31
Number of tRNA	90	87	88	87	82
Number of rRNA	32	27	30	25	12

**Table 2:** Expression changes of the heat shock protein genes in T70 vs. T60 treatment.

Gene ID	Gene	Description	Fold Change
gene0064		Heat shock protein 33 (molecular chaperonin), a cytoplasmically localized protein	2.3
gene0701	*clpB*	Unfold insoluble protein aggregates, and co-factor of Hsp70/DnaK	2.8
gene1200	*clpP*	Clp protease	5.3
gene2328	*dnaJ*	Heat shock protein 40, co-factor of Hsp70	2.8
gene2329	*dnaK*	Folding and unfolding protein, providing thermotolerance on extreme environment	2.7
gene2330	*grpE*	Encoding protein GrpE, HSP-70 cofactor	4.5
gene2447	*mreB*	Rod shape-determining protein MreB	3.1
gene2482	*clpX*	ATP-dependent Clp protease	2.1

## Supplementary Materials

The following are available online at https://www.mdpi.com/2076-2607/8/9/1357/s1, Figure S1. Colony morphology and microscopic observation of the B5 strain; Figure S2. Phylogenetic tree based on the alignment of the recN gene sequence of strain B5; Figure S3. The utilization of different carbon sources of G. stearothermophilus B5; Figure S4. Gene count distributions of carbohydrate-active enzyme (CAZy) families of G. stearothermophilus B5; Table S1. The utilization of different carbon sources for *G. stearothermophilus* B5; Table S2. COG annotation of *G. stearothermophilus* B5; Table S3. The gene expression of CAZymes in *G. stearothermophilus* B5; Table S4. The heat-response sensitive genes in *G. stearothermophilus* B5; Table S5. The significant differentially expressed genes between different treatments; Table S6. The up-regulated genes related to ribosome pathway.
