# Supplementary material for: Genomic and Transcriptome Analyses of a Thermophilic Bacterium Geobacillus stearothermophilus B5 Isolated from Compost Reveal Its Enzymatic Basis for Lignocellulose Degradation
Source: Microorganisms. 2020 Sep 4;8(9):1357. doi: 10.3390/microorganisms8091357 (PMC7564440; doi:10.3390/microorganisms8091357)
Supplement: Supplementary file 1 [file microorganisms-08-01357-s001.zip › Supplementary/Supplementary Figures.docx]

Article

Genomic and transcriptome analyses of a thermophilic bacterium *Geobacillus stearothermophilus* B5 isolated from compost reveal its enzymatic basis for lignocellulose degradation

**Mengmeng Wang^1^, Jiaxi Miao^1^, Xuanqing Wang^1^, Tuo Li^1^, Han Zhu^1^, Dongyang Liu^1^*, Qirong Shen^1^**

Jiangsu Provincial Key Lab of Solid Organic Waste Utilization, Jiangsu Collaborative Innovation Center of Solid Organic Wastes, Educational Ministry Engineering Center of Resource-saving fertilizers, Nanjing Agricultural University, Nanjing 210095, Jiangsu, Peoples Republic of China

**Email addresses:**

Mengmeng Wang: 2013203040@njau.edu.cn

Jiaxi Miao: 2017203039@njau.edu.cn

Xuanqing Wang: 2017203031@njau.edu.cn

Tuo Li: 2018203039@njau.edu.cn

Han Zhu: 2018103120@njau.edu.cn

Dongyang Liu: liudongyang@njau.edu.cn

Qirong Shen: [shenqirong@njau.edu.cn](mailto:shenqirong@njau.edu.cn)

*****Correspondence: liudongyang@njau.edu.cn; Tel: +86 25 84396853; Fax: +86 25 84396853, College of Resources and Environmental Science, Nanjing Agricultural University, Nanjing, CHINA


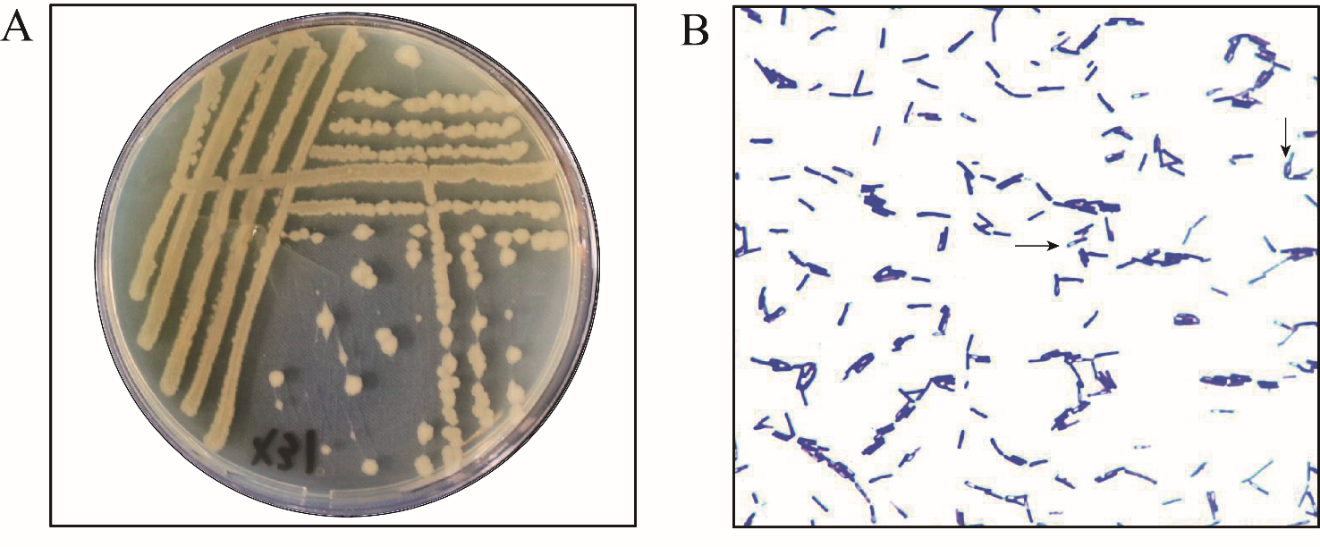


**Figure S1.** Colony morphology and microscopic observation of the B5 strain

**
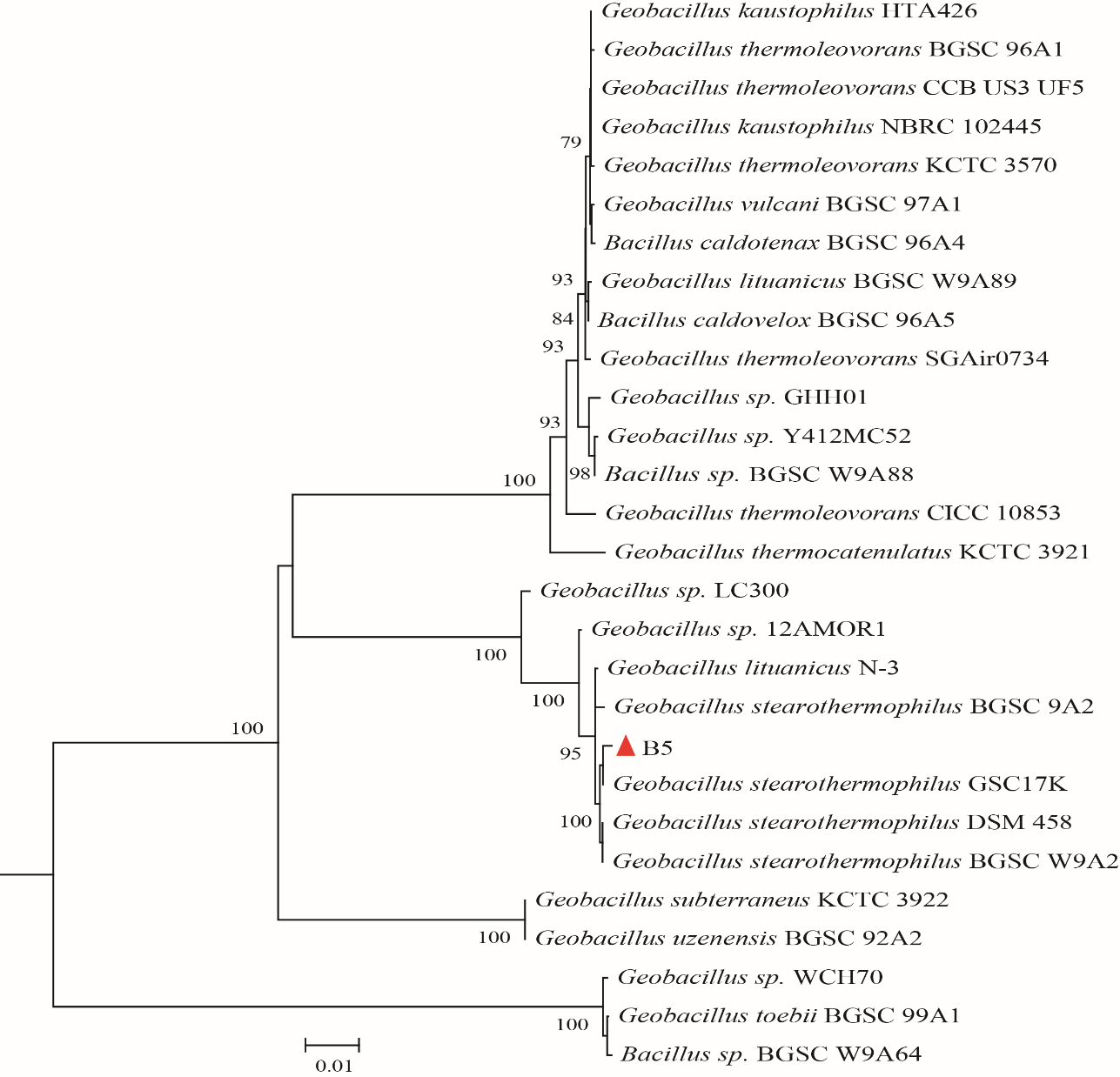
 Fig. S2**

**Figure. S2** Phylogenetic tree based on the alignment of the *recN* gene sequence of strain B5


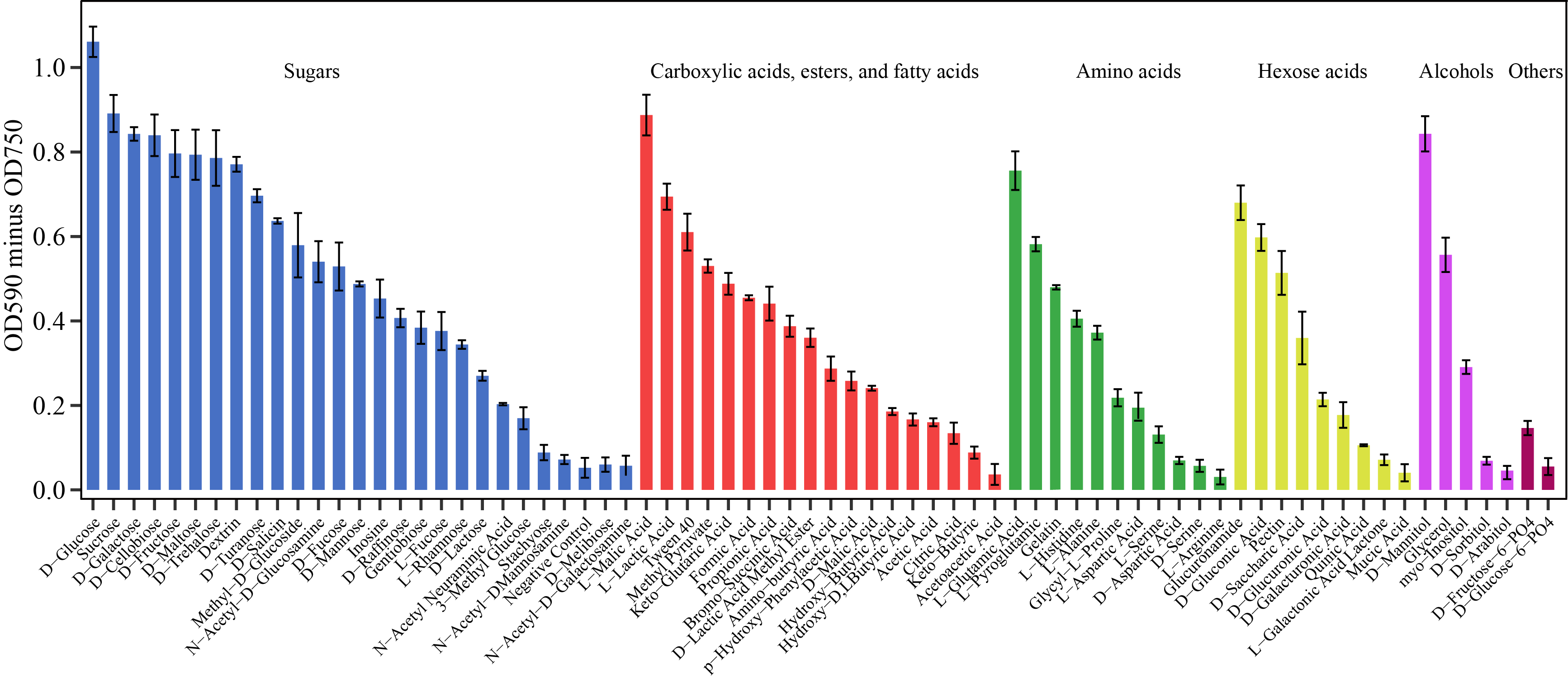


**Figure. S3** The utilization of different carbon sources of *G. stearothermophilus* B5


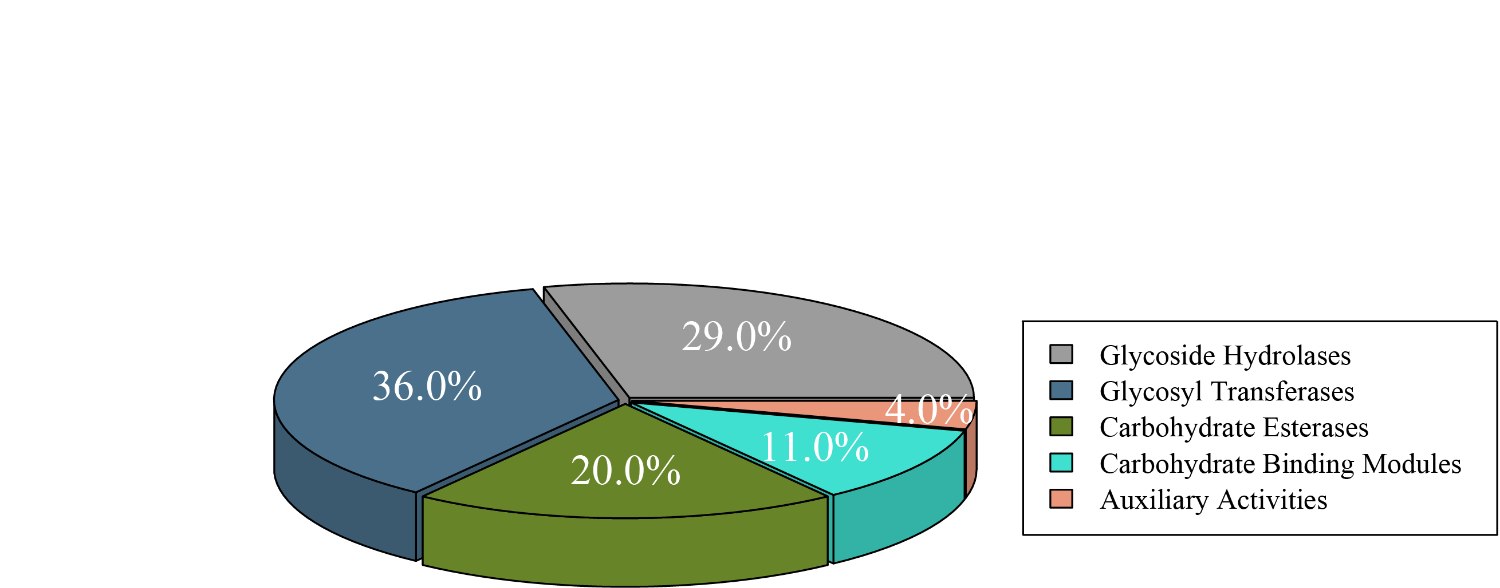


**Fig. S4**

**Figure. S4** Gene count distributions of carbohydrate-active enzyme (CAZy) families of *G. stearothermophilus* B5.
